# Draft Genome Sequences of Two Antimicrobial Compound-Producing Strains of *Bacillus* Species, TM-R and SY1-1

**DOI:** 10.1128/mra.01010-21

**Published:** 2022-02-03

**Authors:** Noriko Ryuda, Takashi Someya, Yukio Nagano

**Affiliations:** a Analytical Research Center for Experimental Sciences, Saga University, Saga, Japan; b Faculty of Agriculture, Saga University, Saga, Japan; Indiana University, Bloomington

## Abstract

Two antimicrobial compound-producing strains of *Bacillus* species, namely, TM-R and SY1-1, have been identified previously. In this study, we report the draft genome sequences of these strains and demonstrate the presence of 12 and 14 gene clusters for secondary metabolite biosynthesis in TM-R and SY1-1, respectively.

## ANNOUNCEMENT

Two antimicrobial compound-producing strains of *Bacillus* species have been characterized (1, 2). The TM-R strain was isolated as a lipolytic bacterium from a restaurant grease trap and suppresses the growth of five of the six studied fungi and produces volatile organic compounds with antifungal activity (1). The 16S rRNA gene analysis did not reveal whether this strain belonged to B. pumilus or B. altitudinis; however, it was categorized as *B. pumilus* based on its biochemical characterization. The SY1-1 strain was isolated in cow manure compost amended with wild grass in the Aso region of Kumamoto, Japan, and produces antimicrobial compounds that inhibit the growth of both fungi and bacteria (2). The 16S rRNA gene analysis did not reveal whether this strain belonged to B. amyloliquefaciens or B. velezensis. Here, we report the draft genome sequences of these strains.

The bacteria were cultured on nutrient agar at 30°C for 24 h aerobically, and their colonies were scraped from the plate using glass beads. Genomic DNA was isolated using a NucleoSpin microbial DNA kit (Macherey-Nagel, Duren, Germany) according to the manufacturer’s protocol. Sequencing libraries were prepared using the NEBNext Ultra DNA library prep kit for Illumina (New England Biolabs [NEB], USA). The libraries were sequenced with 150-bp paired-end reads by Novogene (Beijing, China) using a NovaSeq 6000 instrument (Illumina, San Diego, CA, USA). Adapter sequences and low-quality bases were trimmed using fastp v 0.20.1 with default parameters (e.g., cut_mean_quality = 20) (3). Genome assembly was performed using SPAdes v 3.15.3 (4), and sequences of less than 200 bp were excluded. Gene annotation and taxonomy checks were performed using DFAST v 1.2.13 (https://dfast.ddbj.nig.ac.jp) by selecting “perform taxonomy check,” which calculates average nucleotide identity (5). Gene clusters for secondary metabolite biosynthesis were predicted using antiSMASH v 6.0.1 by selecting “relaxed” as the detection strictness as well as all extra features (6). Default parameters were used for all tools unless otherwise stated.

Table 1 shows the results of the genome assemblies. Taxonomic prediction showed that TM-R was a *B. altitudinis* strain, whereas SY1-1 was a *B. velezensis* strain. The antiSMASH analysis showed that TM-R has 12 gene clusters for secondary metabolite biosynthesis—4 nonribosomal peptide synthetase clusters (NRPS), 2 RRE-element-containing clusters, 2 beta-lactones (beta-lactone containing protease inhibitor), 1 terpene, 1 terpene/siderophore (siderophore cluster), 1 type III polyketide synthase (T3PKS), and 1 unspecified ribosomally synthesized and posttranslationally modified peptide product (RiPP)-like cluster. Furthermore, the analysis showed that SY1-1 has 14 gene clusters for secondary metabolite biosynthesis—2 NRPS, 2 trans-AT polyketide synthases (transAT-PKS), 2 terpenes, 1 T3PKS, 1 PKS-like (other types of PKS cluster), 1 transAT-PKS/T3PKS/NRPS, 1 NRPS/transAT-PKS/beta-lactone, 1 NRPS/RiPP-like, 1 lantipeptide-class-II (class II lanthipeptide clusters like mutacin II), 1 ladderane (ladderane cluster), and 1 other (cluster containing a secondary metabolite-related protein that does not fit into any other category). Most of these metabolites may be responsible for the production of antimicrobial compounds.

**TABLE 1:** Assembly metrics and annotated features of two *Bacillus* strains

Strain	Predicted species name	No. of filtered reads	Total sequence length (bp)	No. of contigs	Avg coverage (×)	*N*_50_ (bp)	No. of CDSs^*a*^	G+C content (%)	GenBank accession no.	SRA accession no.
TM-R	*B. altitudinis*	5,470,534	3,778,259	32	529	905,837	3,872	41.2	BPWB01000000	DRA012762
SY1-1	*B. velezensis*	6,667,552	4,135,505	41	396	1,158,586	4,119	46.1	BPWC01000000	DRA012763

a CDSs, coding DNA sequences.

In conclusion, the draft genome sequences provide fundamental information for the identification of antimicrobial compounds.

### Data availability.

The draft genome sequences and corresponding read data are available in DDBJ/GenBank. The DDBJ/GenBank and SRA accession numbers for TM-R and SY1-1 are listed in Table 1.

## References

[1] Morita T, Tanaka I, Ryuda N, Ikari M, Ueno D, Someya T. 2019. Antifungal spectrum characterization and identification of strong volatile organic compounds produced by *Bacillus pumilus* TM-R. Heliyon 5:e01817. doi:10.1016/j.heliyon.2019.e01817. 31206088 PMC6558263

[2] Ryuda N, Sueishi Y, Koga Y, Sakamoto Y, Mitani K, Abe H, Ueno D, Someya T. 2021. Distribution and characteristics of antagonistic bacteria in grass manure and grass manure amended arable soil in the Aso region of Kumamoto. Jpn Soil Microorg 75:70–78. (In Japanese.) doi:10.18946/jssm.75.2_70.

[3] Chen S, Zhou Y, Chen Y, Gu J. 2018. fastp: an ultra-fast all-in-one FASTQ preprocessor. Bioinformatics 34:i884–i890. doi:10.1093/bioinformatics/bty560. 30423086 PMC6129281

[4] Prjibelski A, Antipov D, Meleshko D, Lapidus A, Korobeynikov A. 2020. Using SPAdes de novo assembler. Curr Protoc Bioinformatics 70:e102. doi:10.1002/cpbi.102. 32559359

[5] Tanizawa Y, Fujisawa T, Nakamura Y. 2018. DFAST: a flexible prokaryotic genome annotation pipeline for faster genome publication. Bioinformatics 34:1037–1039. doi:10.1093/bioinformatics/btx713. 29106469 PMC5860143

[6] Blin K, Shaw S, Kloosterman AM, Charlop-Powers Z, van Wezel GP, Medema MH, Weber T. 2021. antiSMASH 6.0: improving cluster detection and comparison capabilities. Nucleic Acids Res 49:W29–W35. doi:10.1093/nar/gkab335. 33978755 PMC8262755

